# Distinction of IgG4-related mastitis from breast cancer: a case report

**DOI:** 10.1186/s40792-019-0681-y

**Published:** 2019-07-31

**Authors:** Banri Tsuda, Nobue Kumaki, Rie Ishida, Mari Mizuno, Kozue Yokoyama, Risa Oshitanai, Mayako Terao, Toru Morioka, Takuho Okamura, Yuki Saito, Yasuhiro Suzuki, Naoki Niikura

**Affiliations:** 1 Department of Breast and Endocrine Surgery, Tokai University School of Medicine, 143 Shimokasuya, Isehara, Kanagawa 259-1193 Japan; 2 Department of Pathology, Tokai University School of Medicine, Kanagawa, Japan; 3 Department of Breast and Endocrine Surgery, Tokai University Hachioji Hospital, Hachioji, Japan

**Keywords:** IgG4-related sclerosing disease, IgG4-related mastitis, Breast cancer, Steroid therapy

## Abstract

**Background:**

Immunoglobulin (Ig) G4-related sclerosing disease is a pathological concept proposed in Japan during the early 2000s. This lesion-forming disease may exhibit characteristics of a systemic disease but often affects a single organ. To date, IgG4-related sclerosing disease in the mammary gland, or IgG4-related mastitis (IgG4-RM), has rarely been reported.

**Case presentation:**

Here, we describe the case of a female patient who was admitted to our hospital with the main complaints of left breast and axillary lymphadenopathy. A careful diagnostic imaging examination led to an initial suspicion of breast cancer. However, a needle biopsy led to a diagnosis of IgG4-RM. Subsequently, the patient was successfully treated with predonin.

**Conclusions:**

The treatment requirements for breast cancer and IgG4-RM differ considerably. This is a good example of a case wherein unnecessary surgical treatment, which is indicated for breast cancer, was avoided by needle biopsy. Accordingly, the patient was appropriately treated with steroids following a correct diagnosis.

## Background

The concept of immunoglobulin (Ig) G4-related sclerosing disease was recently proposed in Japan to explain the similar pathological imaging findings and responses to steroid therapy between extra-pancreatic lesions associated with autoimmune pancreatitis and pancreatic pathologies associated with IgG4-positive plasma cell infiltration [1]. Umbara et al. first proposed comprehensive diagnostic criteria for IgG4-related sclerosing disease in 2011 [2]; these include high-density lymphoplasmic cell invasion, fibrosis, and obliterative phlebitis. Although IgG4-related sclerosing disease may exhibit characteristics of a systemic disease, it often remains restricted to a single organ [2] [3]. To date, approximately 8000 patients with IgG4-related sclerosing disease have been reported [4]. The peak age of onset occurs during the seventh decade of life [5], and the cause remains unknown [6].

Although IgG4-related sclerosing diseases occur in various organs, these lesions are rarely observed in the mammary glands [5, 7]. In this report, we describe a case of IgG4-related mastitis (IgG4-RM) requiring differentiation from breast cancer.

## Case presentation

A 70-year-old woman initially presented in the Department of Blood Medicine at our hospital with a complaint of reactive axillary lymphadenopathy 6 years earlier. Subsequently, a multiple nodule in the left breast was observed during a recent computed tomography follow-up, at which time the patient was introduced to our department. Her chief complaint involved mild left axillary pain. She had no notable personal or family history.

**Fig. 1:**
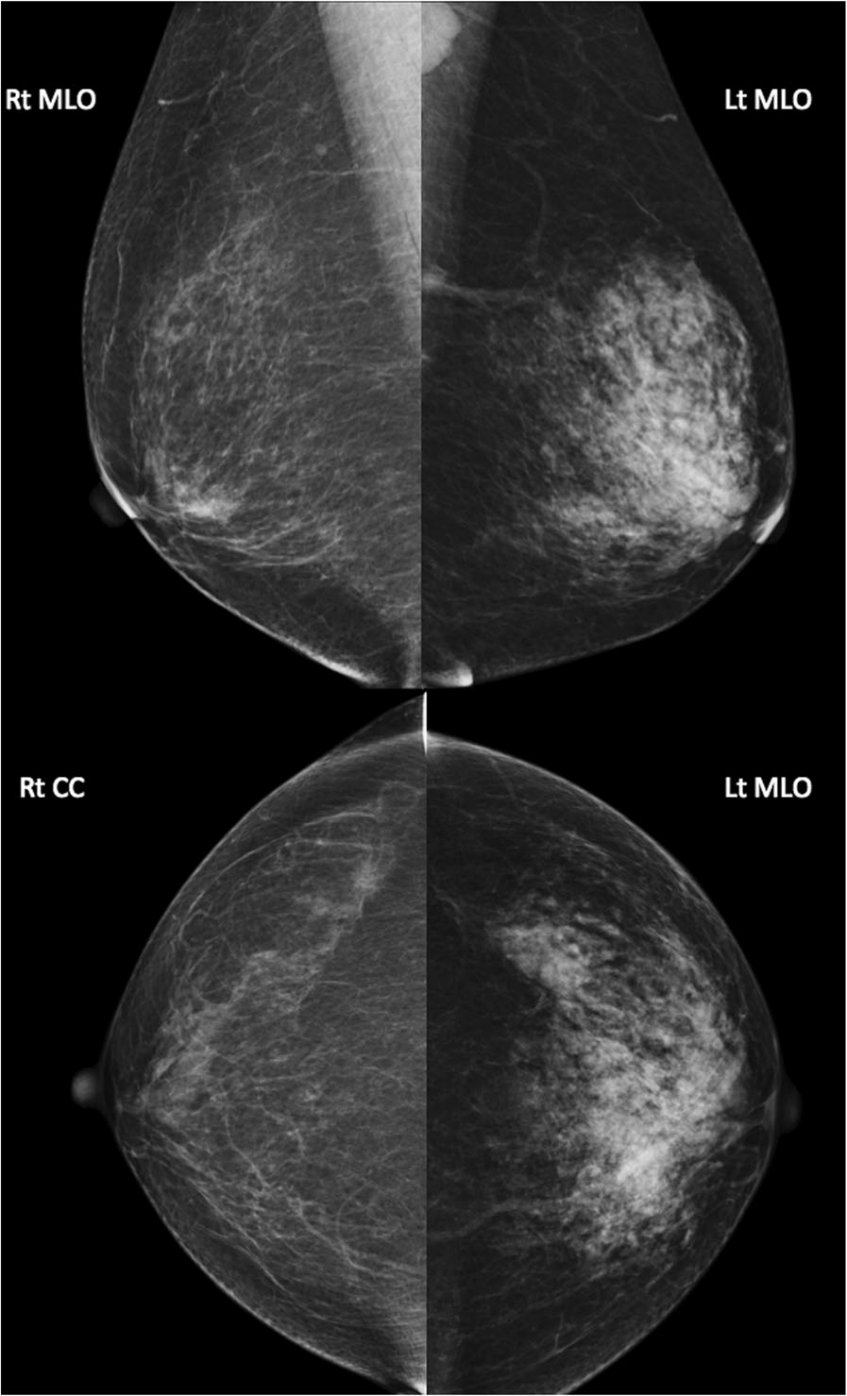
Mammography findings. Medio-lateral oblique (MLO) and cranio-caudal (CC) images are shown. No apparent tumor shadow or calcification was observed in the bilateral mammary gland tissues. However, obvious differences were observed between the left and right sides, leading to a diagnosis of asymmetric breast tissue (category 3). In the left breast MLO, there is an axillary lymph node which seems to be reactive swelling

**Fig. 2:**
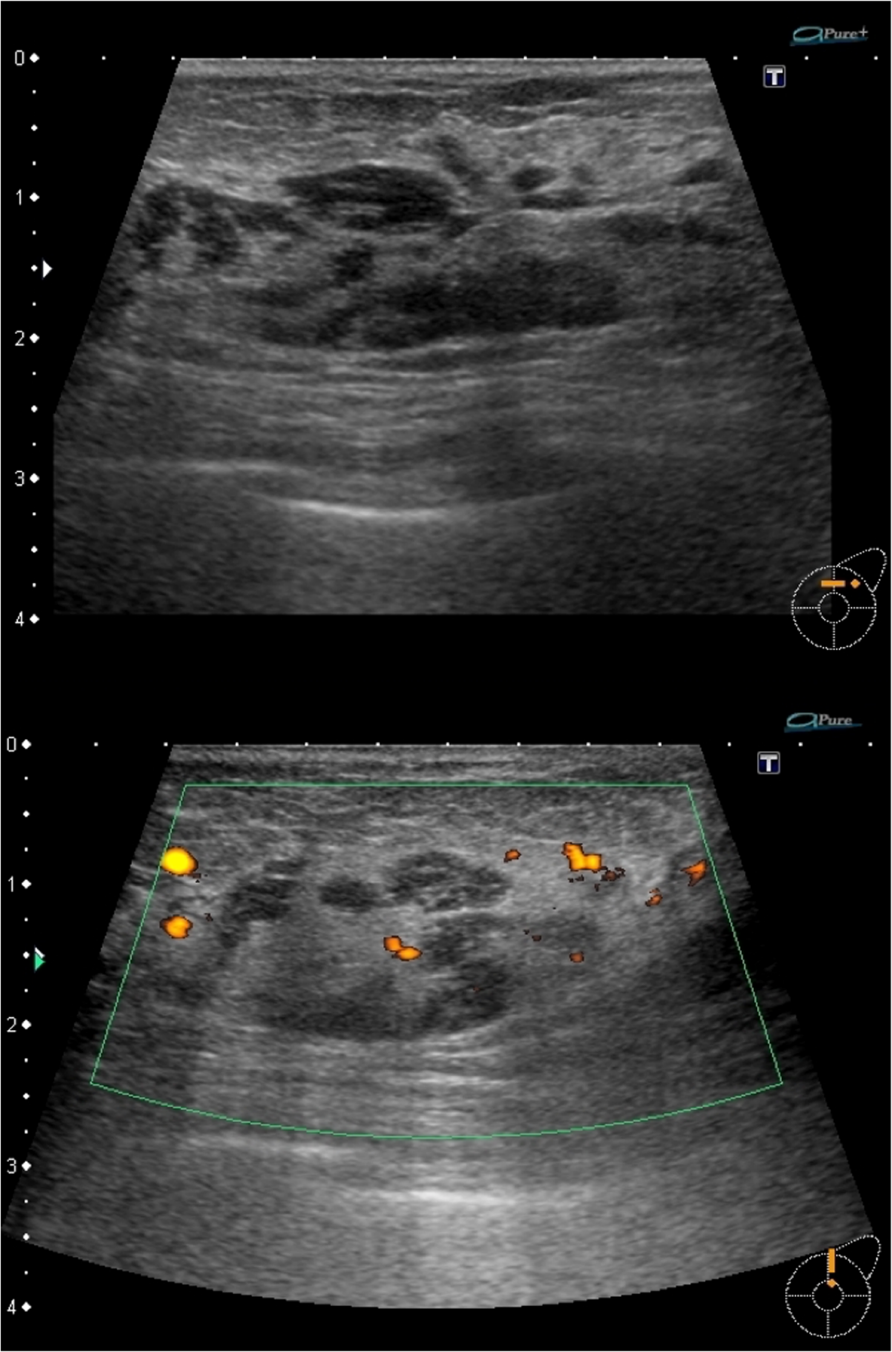
Ultrasonography of the left breast. The C region of the left breast contained an irregularly shaped, poorly echoic region. Large- and small-sized irregularities were observed over a wide range that excluded the B region. The mammary gland tissues clearly differed in both properties and thickness between the left and right sides. A poor blood flow signal was observed on Doppler ultrasonography. Swelling and thickness were observed in several axillary lymph nodes; however, the cortical echo level was low and internal heterogeneity was observed mostly using the lymph node gate; this finding was not one-sided

**Fig. 3:**
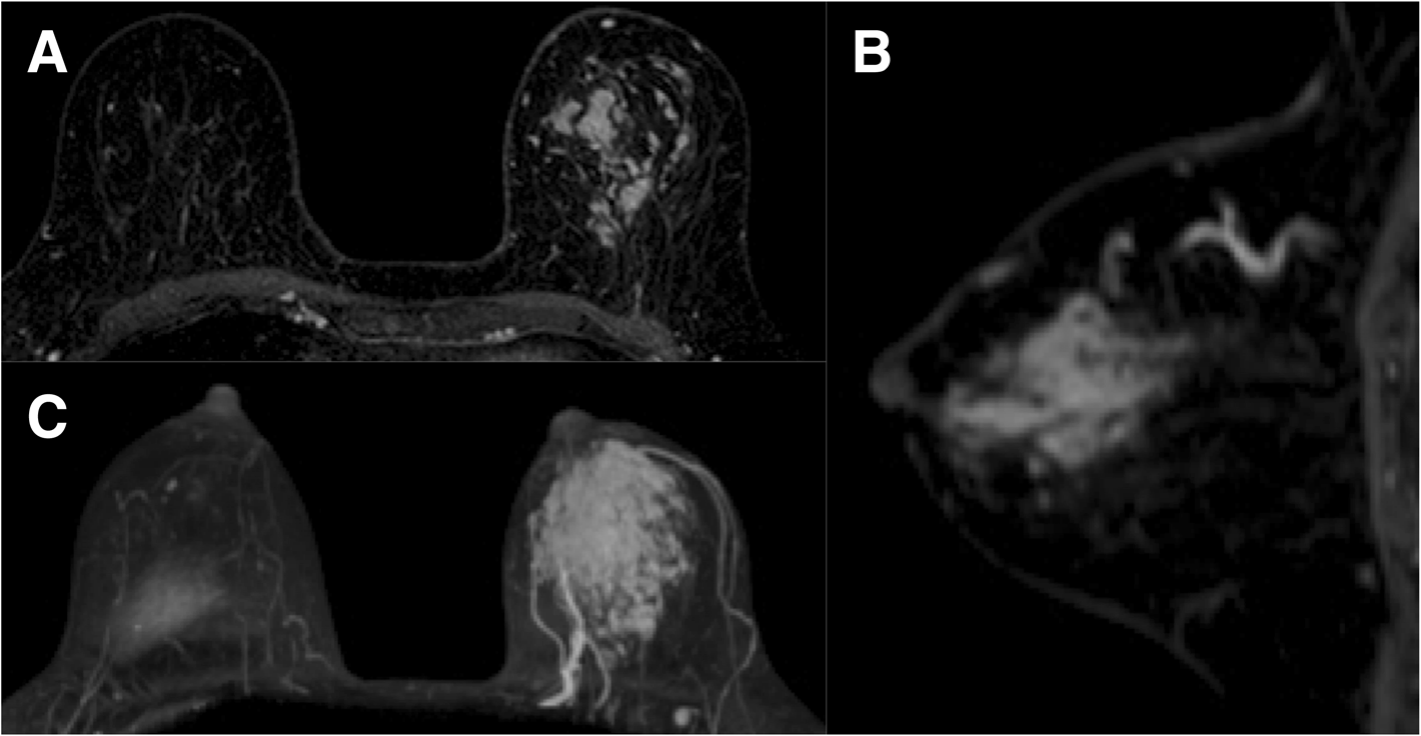
Findings from an enhanced magnetic resonance imaging scan of the breast. **a**, **b** Fat suppression. **c** MIP. Numerous punctate areas of contrast with dynamic washout were detected throughout the left breast. No invasion of the pectoral muscle, chest wall, or skin was observed

During the physical examination, we palpated a soft, elastic, and egg-sized tumor with a smooth surface in the left breast upper region. This tumor exhibited mild tenderness. Additionally, we palpated multiple bean-sized tumors in the left axilla. The fusion status was unclear. Following mammography, the right and left mammary tissues were classified as categories 1 and 3, respectively, and the patient was diagnosed with asymmetric mammary gland tissue (Fig. 1). Ultrasonography of the upper-outer area of the left breast revealed a poorly echoic region in which irregularly sized and shaped abnormalities were observed over a wide range of the breast, excluding the B region (Fig. 2). Obvious differences in tissue properties and thicknesses were observed between the left and right breasts. Doppler ultrasonography revealed a poor blood flow signal. Magnetic resonance imaging revealed numerous punctate areas of contrast with dynamic phase washout throughout the left breast, leading to a suspicion of breast cancer. No invasion of the pectoral muscle, chest wall, and skin was observed (Fig. 3).

Given the examination findings, which differed from typical breast cancer, the differential diagnosis also included malignant lymphoma. Frequent bilateral cervical, supraclavicular fossa, axillary, and inguinal lymphadenopathies were observed. Many of these areas were thick, with a low cortical echo level and internal heterogeneity. Most of these abnormalities had lymph node gates, and no deviation of the lymph nodes was observed.

**Fig. 4:**
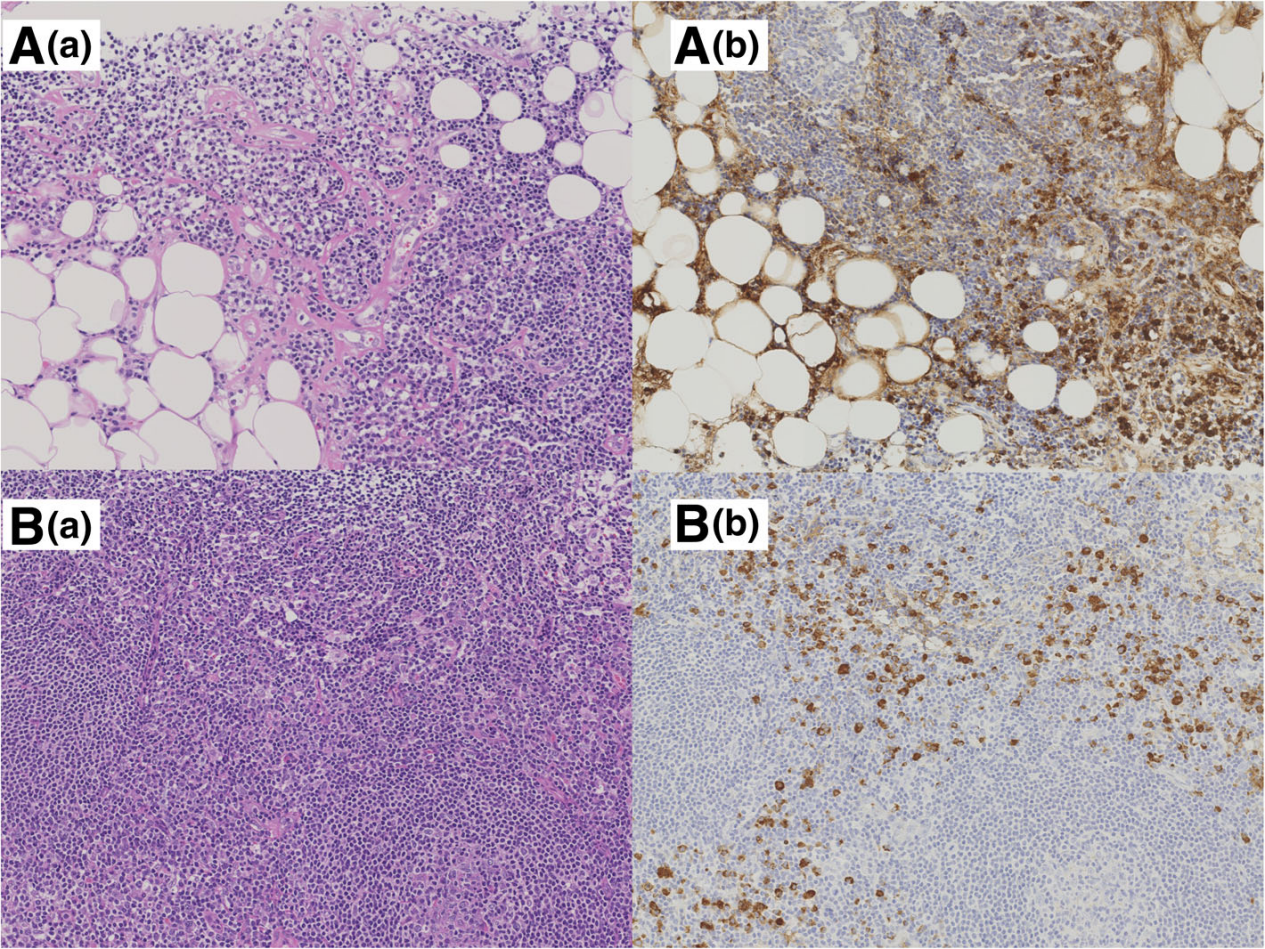
Pathological findings from a core needle biopsy specimen after hematoxylin and eosin-staining and immunohistochemical staining for immunoglobulin (Ig) G4. **a** left breast. (a) Significant lymphocytic and plasma cell infiltration and interstitial fibrosis were observed. (b) More than 40% of the IgG-positive plasma cells were IgG4-positive. The mammary tissue contained almost no glandular component. **b** left axial lymph node. (a) Many plasma cells were observed in an expansive interfollicular area. (b) Many IgG4-positive plasma cells were observed

A core needle biopsy of the left breast tissue revealed a high degree of mixed T and B lymphocytic and plasma cell infiltration, as well as interstitial fibrosis (Fig. 4A). Mammary glands were rarely seen. A core needle biopsy of the left axillary lymph node showed expansion of the interfollicular area, and these expanded areas contained conspicuous plasma cells (Fig. 4B) with no Ig light chain restriction detected upon in in situ hybridization. Many plasma cells were IgG-positive, and more than 40% were IgG4-positive. These biopsy findings led to a diagnosis of IgG4-related mastitis of the left breast. Following diagnosis, the case was transferred to the Department of Internal Medicine–Rheumatology, and the patient was treated with predonin (10 mg/day). During a 4-year follow-up since the diagnosis, no recurrence of the initial lesion has been observed.

As noted, the concept of IgG4-related sclerosing disease was proposed to address the similarities of this condition with those of pathologic pancreatic extracorporeal lesions associated with autoimmune pancreatitis; additionally, both lesions respond well to steroid treatment [1, 8, 9]. Since the introduction of this concept, similar tumorigenic lesions with inflammatory cell infiltration have been found in various organs [10], leading to the worldwide recognition of this group of diseases [8].

The existing comprehensive diagnostic criteria for IgG4-related sclerosing disease include the following items [2]. Clinical findings include diffuse or localized enlargement, mass, nodular, and/or hypertrophic lesions in single or multiple organs. Blood findings include high IgG4emia, defined as a level of ≥ 135 mg/dL. Pathological findings include prominent lymphocytes, plasma cell infiltration, and fibrosis, with an IgG4-positive/IgG-positive cell ratio > 40% and ≥ 10 IgG4-positive plasma cells per high-powered microscopic field. Cases are defined as definite, probable, or possible if all three, clinical and pathological, or clinical and blood criteria are met, respectively.

In the present case, the mammary gland and axillary lymph node findings met the clinical criteria. The IgG4 level was not initially investigated because of the original suspicion of breast cancer; however, this parameter was later examined and found to be 882 mg/dL (reference range 4.5–117 mg/dL), which met the blood criterion. Furthermore, the analytical findings of the mammary gland tissue and lymph node met the pathological criteria. Accordingly, the present case was finally and definitively diagnosed with IgG4-RM.

As noted, breast cancer was initially suspected, and IgG4-RM was diagnosed only after a needle biopsy. The diagnosis may have been reached sooner if an IgG4 analysis had been performed. However, this is not a realistic assumption. Still, in such cases, misdiagnosis can complicate treatment. Currently, surgery is the standard treatment for breast cancer, whereas IgG4-RM responds well to steroid therapy and does not tend to recur [11]. Therefore, a detailed histopathological analysis of a needle biopsy specimen could avoid an unnecessary surgical procedure.

## Conclusions

IgG4-RM is very rare, and the clinical and imaging findings often lead to a suspicion of breast cancer. In such cases, a histopathological examination by a pathologist familiar with pathological conditions of the mammary gland is important to ensure a correct diagnosis and the avoidance of unnecessary surgical procedures.

## Data Availability

Not applicable.

## Abbreviations

Ig: Immunoglobulin
RM: Related mastitis

## References

[1] Kamisawa T, Okamoto A (2006). Autoimmune pancreatitis: proposal of IgG4-related sclerosing disease. J Gastroenterol.

[2] Umehara H, Okazaki K, Masaki Y, Kawano M, Yamamoto M, Saeki T (2012). A novel clinical entity, IgG4-related disease (IgG4RD): general concept and details. Mod Rheumatol.

[3] Cheuk W, Yuen HK, Chu SY, Chiu EK, Lam LK, Chan JK (2008). Lymphadenopathy of IgG4-related sclerosing disease. Am J Surg Pathol.

[4] Cheuk W, Chan AC, Lam WL, Chow SM, Crowley P, Lloydd R (2009). IgG4-related sclerosing mastitis: description of a new member of the IgG4-related sclerosing diseases. Am J Surg Pathol.

[5] Bateman AC, Deheragoda MG (2009). IgG4-related systemic sclerosing disease - an emerging and under-diagnosed condition. Histopathology.

[6] Zen Y, Kasahara Y, Horita K, Miyayama S, Miura S, Kitagawa S (2005). Inflammatory pseudotumor of the breast in a patient with a high serum IgG4 level: histologic similarity to sclerosing pancreatitis. Am J Surg Pathol.

[7] Kamisawa T, Funata N, Hayashi Y, Eishi Y, Koike M, Tsuruta K (2003). A new clinicopathological entity of IgG4-related autoimmune disease. J Gastroenterol.

[8] Kamisawa T, Okamoto A (2008). IgG4-related sclerosing disease. World J Gastroenterol.

[9] Chougule A, Bal A, Das A, Singh G (2015). IgG4 related sclerosing mastitis: expanding the morphological spectrum of IgG4 related diseases. Pathology.

[10] Detlefsen S, Drewes AM (2009). Autoimmune pancreatitis. Scand J Gastroenterol.

[11] Dhobale S, Bedetti C, Killian P, Ilyas M, Liput J, Jasnosz K (2009). IgG4 related sclerosing disease with multiple organ involvements and response to corticosteroid treatment. J Clin Rheumatol.

